# ASPP2 Links the Apical Lateral Polarity Complex to the Regulation of YAP Activity in Epithelial Cells

**DOI:** 10.1371/journal.pone.0111384

**Published:** 2014-10-31

**Authors:** Christophe Royer, Sofia Koch, Xiao Qin, Jaroslav Zak, Ludovico Buti, Ewa Dudziec, Shan Zhong, Indrika Ratnayaka, Shankar Srinivas, Xin Lu

**Affiliations:** 1 Ludwig Institute for Cancer Research, Nuffield Department of Clinical Medicine, University of Oxford, Oxford, United Kingdom; 2 Department of Physiology, Anatomy and Genetics, University of Oxford, Oxford, United Kingdom; Institute of Molecular and Cell Biology, Biopolis, United States of America

## Abstract

The Hippo pathway, by tightly controlling the phosphorylation state and activity of the transcription cofactors YAP and TAZ is essential during development and tissue homeostasis whereas its deregulation may lead to cancer. Recent studies have linked the apicobasal polarity machinery in epithelial cells to components of the Hippo pathway and YAP and TAZ themselves. However the molecular mechanism by which the junctional pool of YAP proteins is released and activated in epithelial cells remains unknown. Here we report that the tumour suppressor ASPP2 forms an apical-lateral polarity complex at the level of tight junctions in polarised epithelial cells, acting as a scaffold for protein phosphatase 1 (PP1) and junctional YAP via dedicated binding domains. ASPP2 thereby directly induces the dephosphorylation and activation of junctional YAP. Collectively, this study unearths a novel mechanistic paradigm revealing the critical role of the apical-lateral polarity complex in activating this localised pool of YAP *in vitro*, in epithelial cells, and *in vivo*, in the murine colonic epithelium. We propose that this mechanism may commonly control YAP functions in epithelial tissues.

## Introduction

The Hippo pathway, by regulating the phosphorylation state and activity of YAP and TAZ (YAP/TAZ), has emerged as a crucial regulator of cell number and differentiation [1]. This is illustrated by recent findings that the Hippo pathway is central to tissue regeneration and the control of organ size. The Hippo pathway must therefore be able to sense global changes in tissue architecture. Interestingly, at the cellular level, the Hippo pathway can integrate signals through several routes, including GPCR signalling [2], mechanical forces [3] and apicobasal polarity [4]. The Hippo pathway is therefore at an ideal position to sense structural changes *in vivo* and consequently regulate these biological processes.

Originally uncovered in Drosophila, the core hippo signalling cascade in mammals is well established [1]: MST1/2 kinases phosphorylate Lats1/2 which subsequently phosphorylate the transcription cofactors YAP/TAZ to induce their cytoplasmic retention or degradation. Thus phosphorylated cytoplasmic YAP/TAZ are unable to bind and activate a variety of transcription factors including TEAD1-4. Interestingly however, several components of the apicobasal polarity machinery, including the Crumb complex, have been shown to interact with YAP/TAZ [5]. In fact an extended list of Hippo pathway regulators contains proteins involved in apicobasal polarity, planar cell polarity and the formation of cell-cell junctions [1]. With the advent of the Hippo interactome, the connection between YAP and junctional and polarity complexes is emerging [6]–[8]. Additionally, in epithelial cells, several positive regulators of the Hippo pathway, such as angiomotin [9], and Hippo pathway components themselves, such as Lats2 [10], co-localise with YAP/TAZ at the apical-lateral domain corresponding to tight junctions. However, how the junctional pool of YAP/TAZ is mobilised and activated by proteins within polarity complexes remains an open question. Despite the wealth of information about the kinases phosphorylating and repressing YAP/TAZ, much less is known about the proteins acting as positive switches. The mammalian Hippo interactome suggests that serine/threonine phosphatases play a key role in regulating the Hippo pathway [6]. Consistently, recent studies showed that protein phosphatase 1 (PP1) dephosphorylates YAP/TAZ *in vitro* [11], [12]. PP1 is also known to interact with the polarity protein Par3 and induce its dephosphorylation *in vitro* [13]. Interestingly, recent evidence suggests that ASPP2, an interacting partner of Par3, YAP and TAZ, that also binds PP1 via its RVxF motif, may promote YAP activity, suggesting that it may link apicobasal polarity to the dephosphorylation of YAP/TAZ [7], [11], [14]–[17].

ASPP2 was originally characterised as an activator of the p53 family of proteins [18], [19]. Recently it was also identified as an important regulator of cell polarity during central nervous system (CNS) development [14]. Mechanistically, ASPP2 regulates apicobasal polarity by interacting with Par3 via its N-terminal coiled-coiled region to form the apical-lateral polarity complex at tight junctions in epithelial cells [14], [15]. The PPxY motif of ASPP2 was reported to interact with the WW domain of YAP *in vitro* [17] and the ASPP2/YAP interaction was confirmed *in vitro* by the recently reported mammalian Hippo interactome studies [6]–[8]. Despite this body of evidence, how ASPP2 mechanistically regulates YAP and the importance of the ASPP2/YAP interaction *in vivo* remain unknown.

To understand how YAP is activated *in vitro* and *in vivo*, in the context of polarised epithelial cells and tissues, we analysed the precise mechanism by which ASPP2 regulates junctional YAP, in parallel in epithelial cell lines and in the colon. Our results reveal the importance of ASPP2 within the apical-lateral polarity complex in linking apicobasal polarity to YAP activity via the recruitment of protein phosphatase 1 and the direct dephosphorylation of junctional YAP.

## Results

### ASPP2 and YAP form a complex at tight junctions

**Figure 1:**
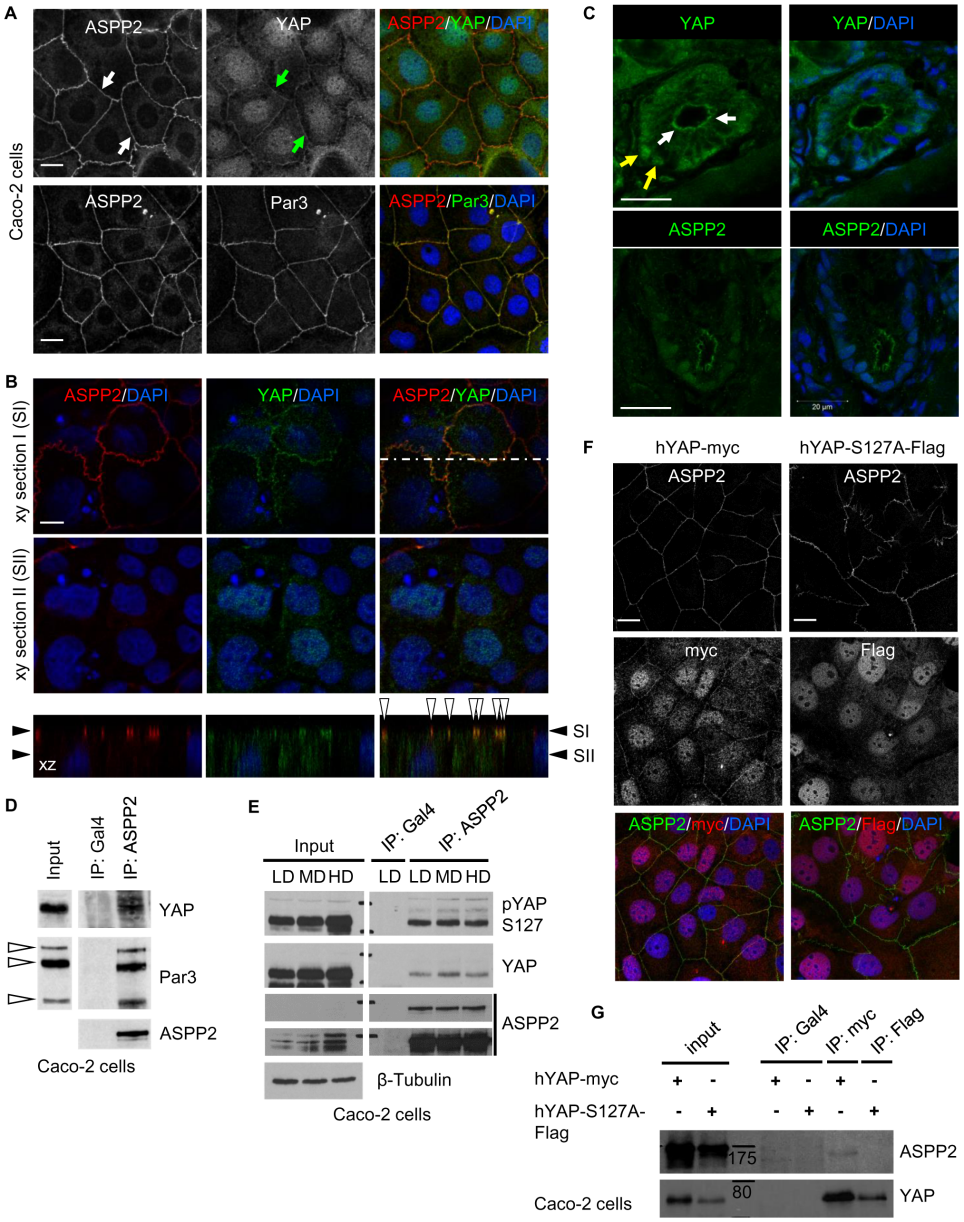
ASPP2 and YAP form a junctional complex in epithelial cells. (**A**) Immunostaining of ASPP2, Par3 and YAP in confluent monolayers of Caco-2 cells. White and green arrows point to tight junction localised ASPP2 and YAP respectively. Scale bars: 20 µm. (**B**) YAP and ASPP2 immunostaining in polarised MDCK cells. Sections SI and SII represent xy optical sections going through the apical-lateral domain and the middle of nuclei respectively. The bottom panel represents the xz section corresponding to the dashed line. SI and SII are shown with black arrowheads. White arrowheads show co-localisation of ASPP2 and YAP at the apical-lateral domain. Nuclei are counterstained with DAPI. Scale bar: 8 µm. (**C**) The localisation of YAP and ASPP2 was analysed by immunostaining of frozen sections obtained from wild type mice. YAP was apical (white arrows) and nuclear (yellow arrows) in the epithelial cells of colonic crypts. Nuclei are counterstained with DAPI. Scale bar: 20 µm. (**D**) The interaction between ASPP2 and Par3 was tested in Caco-2 cells. Endogenous ASPP2 was immunoprecipitated with an anti-ASPP2 mouse monoclonal antibody (DX50.13) and an anti-Gal4 mouse monoclonal antibody was used as a negative control. ASPP2, Par-3 and YAP were detected by SDS-Page/immunoblotting. White arrowheads point to different Par-3 isoforms. (**E**) ASPP2 and YAP co-immunoprecipitation in Caco-2 cells plated at different cell densities. Lysates were obtained from Caco-2 cells plated at various cell densities and endogenous ASPP2 was immunoprecipitated with an anti-ASPP2 mouse monoclonal antibody (DX50.13) and an anti-Gal4 mouse monoclonal antibody was used as a negative control. ASPP2, YAP and YAP phosphorylated at S127 were subsequently detected by SDS-Page/immunoblotting. Long and short exposures are shown for ASPP2. β-tubulin was used as loading control. LD: low density; MD: medium density; HD: high density. (**F-G**) The phosphorylation status of YAP regulates its subcellular localisation and interaction with ASPP2. Stable Caco-2 cells expressing either hYAP-myc or hYAP-S127A-Flag were used to test the requirement of YAP phosphorylation at serine 127 for its interaction with ASPP2 and its junctional localisation. (F) hYAP-myc, hYAP-S127A-Flag and endogenous ASPP2 were detected by immunostaining. DAPI was used to stain nuclei. Scale bars: 20 µm. (G) hYAP-myc and hYAP-S127A-Flag were immunoprecipitated using an anti-myc monoclonal (9E10) and anti-Flag monoclonal antibody respectively. ASPP2 and YAP were subsequently detected by SDS-Page/immunoblotting. Molecular markers are indicated in the figure (values in kDa).

To understand how ASPP2 may control both apicobasal polarity and the activity of YAP, we examined their subcellular localisation in Caco-2 cells, a colorectal cancer cell line exhibiting strong epithelial characteristics and retaining the ability to polarise. Similar to previous observations in epithelial cells and tissues, ASPP2 co-localised with Par3 at tight junctions. Interestingly, in addition to its nuclear localisation, YAP was found to co-localise with ASPP2 at tight junctions, suggesting that they may form a complex at this level (**Figure 1A**). A similar observation could be made in polarised MDCK cells, as YAP co-localised with ASPP2 at the level of the apical-lateral domain where tight junctions reside (**Figure 1B** **and Figure S1A**). In addition, in colonic crypt cells, YAP was also expressed apically towards the lumen, in a localisation pattern reminiscent of ASPP2's, suggesting that, ASPP2 and YAP may also interact at tight junctions *in vivo* (**Figure 1C**). Importantly, reduced junctional and nuclear YAP signal following YAP knockdown could be observed, demonstrating the specificity of the antibody used. Of note, YAP depletion did not affect the localisation of ASPP2 at tight junctions, suggesting that YAP is not important for its subcellular localisation pattern (**Figure S1B**).

Sequence alignments of ASPP1, ASPP2 and iASPP reveal that only ASPP2 contains a WW domain binding PPxY motif within its proline-rich region (**Figure S1C**). Accordingly, ASPP1 was shown not to interact with YAP and neither ASPP1 or iASPP were identified as YAP-binding partners in the Hippo interactome studies [6], [7], [20]. In epithelial cells, ASPP2 co-immunoprecipitated with both Par3 and YAP, further suggesting that endogenous YAP and ASPP2 physically interact at tight junctions, where the majority of ASPP2 resides (**Figure 1D** **and Figure S1D**). Since YAP is regulated in a cell density-dependent manner, we tested whether the ASPP2/YAP interaction could be affected in a similar way. However, ASPP2/YAP complexes could be detected at all densities, suggesting that the ASPP2/YAP interaction is not regulated by cell contact inhibition (**Figure 1E** **and Figure S1D)**. Collectively, these results define an ASPP2/YAP complex at tight junctions in epithelial cells and tissues, indicating that the apical-lateral domain may be the primary site of YAP regulation by ASPP2.

YAP phosphorylation at serine 127 (pYAP S127) is crucial in regulating its subcellular localisation [21], [22]. Interestingly, some pYAP S127 co-localised with ASPP2 at cell-cell junctions (**Figure S1E**) and co-immunoprecipitated with ASPP2 in both Caco-2 and MDCK cells (**Figure 1E** **and Figure S1D**). To test the importance of YAP phosphorylation at S127 in regulating the ASPP2/YAP complex, we used Caco-2 cells stably expressing either wild type (hYAP-myc) or YAP mutated at S127 (hYAP-S127A-Flag). Exogenous wild type YAP adopted a localisation pattern reminiscent of endogenous YAP, partly co-localising with ASPP2 at tight junctions. However, hYAP-S127A-Flag was exclusively nuclear (**Figure 1F**) and was not able to co-immunoprecipitate with ASPP2 (**Figure 1G**). Collectively, these results reveal the importance of YAP phosphorylation at S127 in regulating its interaction with ASPP2 at tight junctions.

### ASPP2 promotes the dephosphorylation of YAP

**Figure 2:**
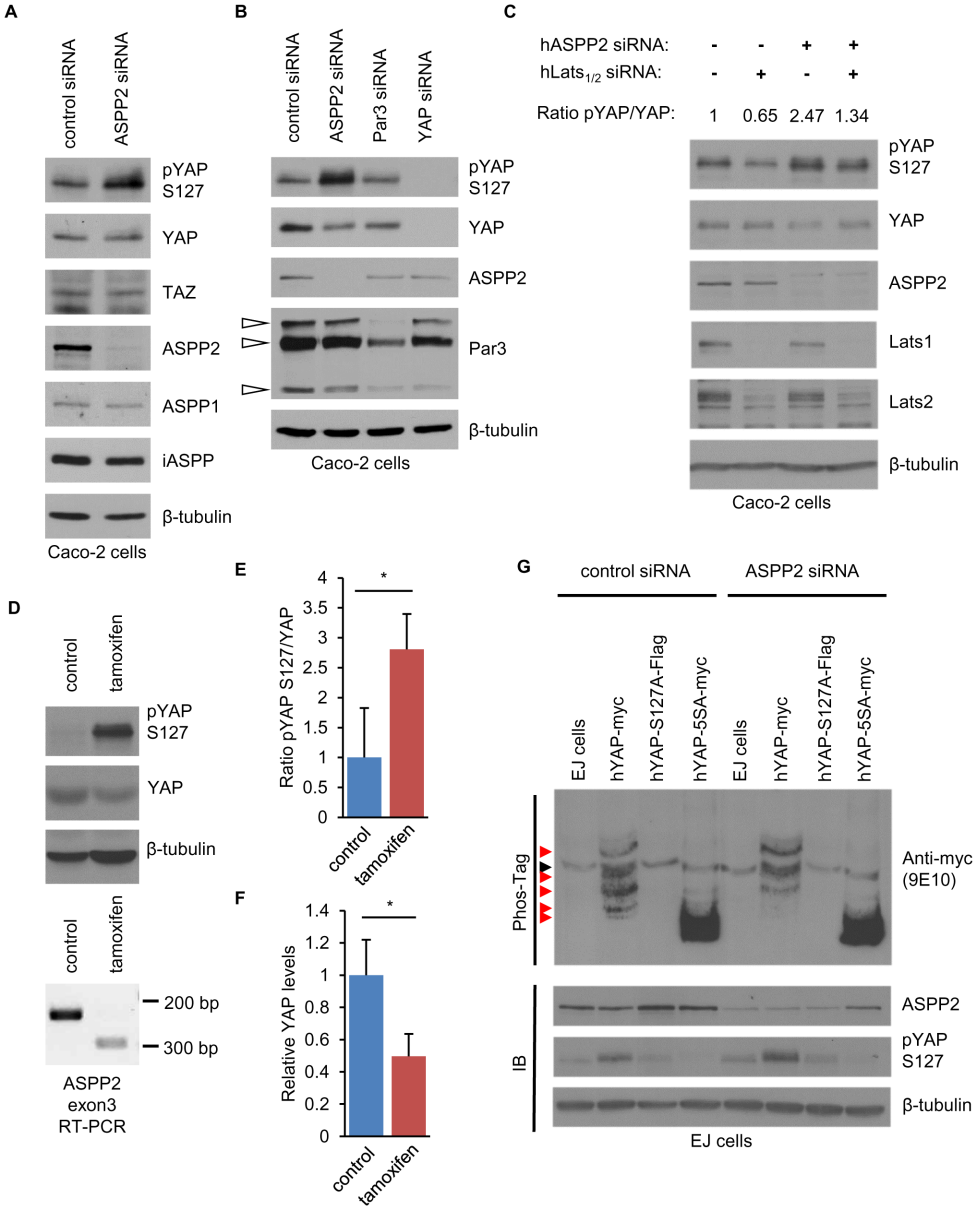
ASPP2 regulates YAP phosphorylation level *in vitro* and *in vivo*. (**A**) ASPP2 depletion leads to increased phosphorylated YAP at serine 127. ASPP2 was depleted in Caco-2 cells using siRNA and SDS-Page/immunoblotting was subsequently performed to analyse the expression level of the indicated proteins. An antibody specifically recognising YAP phosphorylated at serine 127 was used to analyse phosphorylation levels at this particular residue (A-E and G). (**B**) Par3 depletion does not affect the phosphorylation of YAP at S127. SiRNA knockdown was performed to deplete the indicated proteins and their expression was analysed by SDS-Page/immunoblotting. White arrowheads point to different Par-3 isoforms. (**C**) ASPP2 negatively regulates Lats-dependent phosphorylation of YAP at S127. SiRNA against ASPP2 and a combination of siRNA targeting Lats1 and lats2 were used as indicated in the figure. Four days following siRNA knockdown, protein levels were analysed by SDS-Page/immunoblotting. The ratios between pYAPS127 and YAP levels are indicated in the figure. (**D-F**) ASPP2 promotes the dephosphorylation of YAP at S127 in the colon. Exon 3 of *ASPP2* was deleted by injection of tamoxifen in *ASPP2* ^Δ3loxP-CreER^ mice. (D) Upper panel: YAP and pYAP S127 levels were analysed by SDS-Page/immunoblotting using lysates obtained from control and tamoxifen-injected mice (5 controls and 3 tamoxifen-injected animals). β-tubulin was used as loading control. Lower panel: RT-PCR showing deletion of exon3 of ASPP2 following tamoxifen treatment using RNA isolated from the colon of the same mouse. (E-F) Bar graphs representing the ratio between pYAP S127 and total YAP protein levels (E) and YAP protein expression levels normalised by β-tubulin (F). Data was normalised to the control. Error bars indicate standard deviation (*: p<0.05). (**G**) ASPP2 potentially induces the dephosphorylation of YAP at several serine residues. Following ASPP2 knockdown, lysates obtained from EJ cells stably expressing the indicated constructs were analysed by SDS-Page/immunoblotting using Phos-Tag (upper panel) or not (lower panel). Red arrowheads indicate differentially phosphorylated YAP proteins. The black arrowhead points to a non-specific band recognised by the anti-myc antibody (9E10).

Since YAP phosphorylation can affect YAP localisation or stability [21], [23], we investigated the impact of ASPP2 on YAP phosphorylation at S127. In Caco-2 cells, ASPP2 knockdown resulted in a marked increase in pYAP S127 without changes in YAP protein expression level (**Figure 2A**). This was observed in multiple cell lines, including MDCK cells (**Figure S2A**) and cancer cell lines of various epithelial origins (data not shown). Protein expression levels of ASPP1 and iASPP were not altered, suggesting that no compensatory effect occurred (**Figure 2A**). In addition, Par3 knockdown did not influence pYAP S127, further confirming the intrinsic role of ASPP2 (**Figure 2B**). Since YAP is directly phosphorylated by Lats kinases at S127 [21], [24], [25], we tested whether they mediate the increase in pYAP S127 following ASPP2 depletion (**Figure 2C**). As expected, Lats_1/2_ knockdown resulted in the decrease of the ratio between pYAP S127 and total YAP protein level, whereas ASPP2 depletion resulted in its increase. However, when Lats_1/2_ were depleted, ASPP2 depletion did not lead to an increased pYAPS127/YAP ratio, suggesting that this effect is Lats-dependent and that ASPP2 inhibits the Hippo pathway by antagonising Lats-mediated YAP phosphorylation.

Consistently, in the colon of *ASPP2* ^Δexon3^ mice, we observed increased pYAP S127, accompanied by a small decrease in total YAP protein level (**Figure S2B**). We next used *ASPP2* ^Δ3loxP-CreER^ mice to deplete ASPP2 expression in an acute manner using intraperitoneal injections of tamoxifen. Tamoxifen treatments resulted in a significant increase in the pYAPS127/YAP ratio, suggesting that ASPP2 also controls the phosphorylation of YAP at S127 *in vivo* (**Figure 2D and 2E**). Interestingly, as seen in Caco-2 cells four days following ASPP2 knockdown (**Figure 2B and 2C**), this was accompanied by a 2-fold decrease in total YAP protein level, confirming that the regulation of pYAP S127 by ASPP2 may have repercussions on the stability of YAP (**Figure 2D and 2F**).

The degradation of YAP is controlled by its phosphorylation status at several key serine residues [23]. To investigate whether ASPP2 controls additional YAP phosphorylation sites, we used several EJ stable cell lines in a Phos-Tag assay (**Figure 2G**). EJ cells stably expressing wild type YAP displayed up to five bands on a Phos-Tag gel whereas cells expressing a mutant YAP that cannot be phosphorylated [23] only displayed one. Following ASPP2 knockdown in wild type YAP-expressing EJ cells, we observed a shift from fast towards slowly migrating bands, corresponding to an increase in highly phosphorylated forms of YAP. These results indicate that ASPP2 controls the phosphorylation of YAP not only at S127 but also at other serine residues. Together, our results firmly establish ASPP2 as a repressor of YAP phosphorylation and the Hippo pathway.

### ASPP2 binds and dephosphorylates YAP via the recruitment of PP1

**Figure 3:**
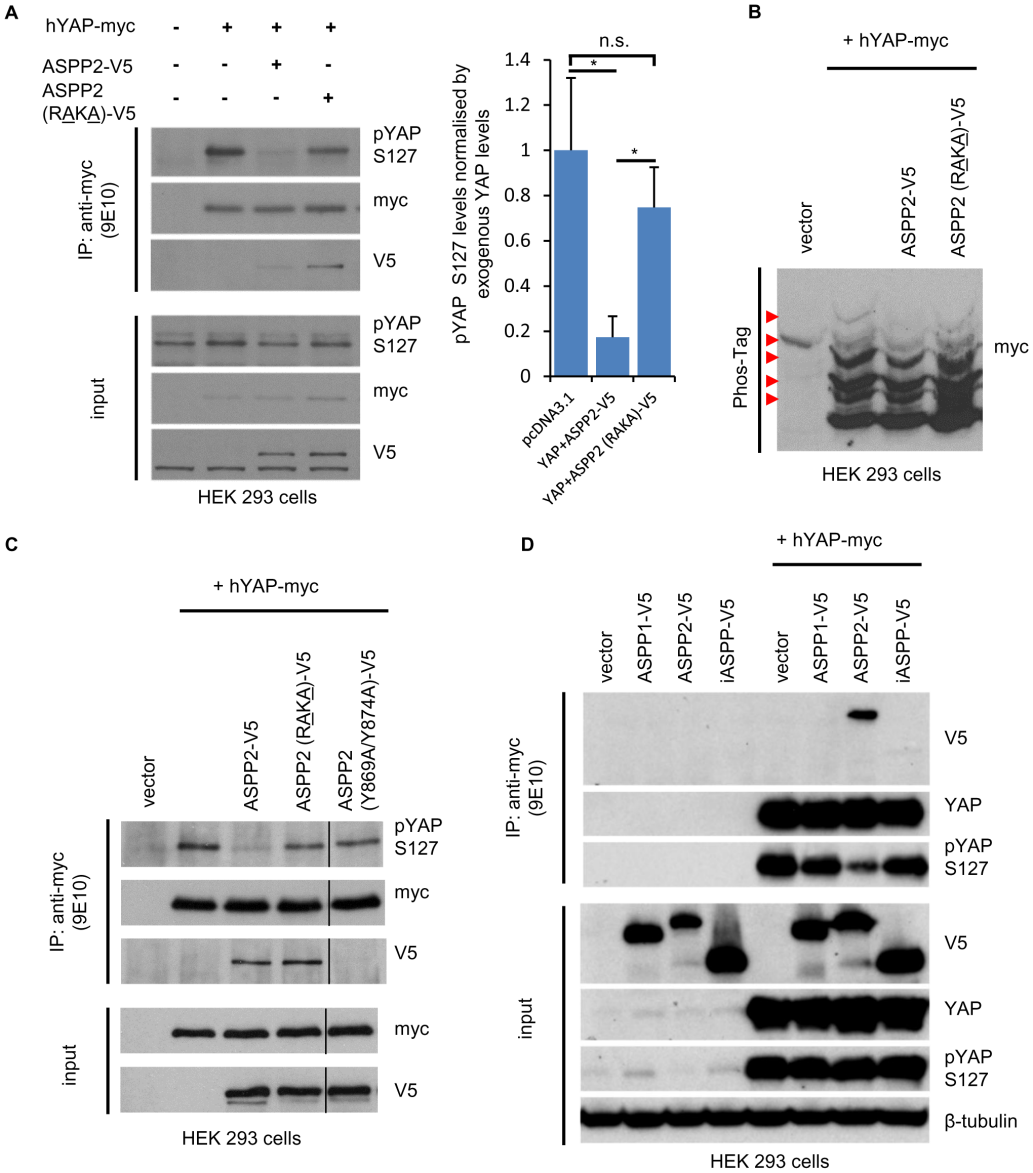
ASPP2 scaffolds PP1 to dephosphorylate YAP. (**A**) The importance of the ASPP2/PP1 interaction in dephosphorylating YAP was tested by transfecting the indicated constructs in HEK293 cells. Exogenous YAP was subsequently immunoprecipitated using an anti-myc mouse monoclonal antibody (9E10) and SDS-Page/immunoblotting was performed using the indicated antibodies. The bar graph represents the ratio between pYAP S127 and total YAP protein levels (n = 3; *: p<0.05; n.s.: non-significant). Error bars indicate standard deviation. (**B**) Lysates obtained from HEK293 cells transfected with the indicated constructs were analysed by SDS-PAGE/immunoblotting on a Phos-Tag gel. (**C**) The ability of ASPP2 (Y869A/Y874A)-V5 to dephosphorylate YAP was tested in HEK293 cells to investigate the importance of the YAP/ASPP2 interaction in this process. Following transfection with the indicated constructs in HEK293 cells, Exogenous YAP was immunoprecipitated using an anti-myc mouse monoclonal antibody (9E10) and SDS-Page/immunoblotting was performed using the indicated antibodies. (**D**) The ability of ASPP2, iASPP and ASPP1-V5 to interact with and dephosphorylate YAP was tested in HEK293 cells. Following transfection with the indicated constructs in HEK293 cells, Exogenous YAP was immunoprecipitated using an anti-myc mouse monoclonal antibody (9E10) and SDS-Page/immunoblotting was performed using the indicated antibodies.

Since ASPP2 can directly bind PP1 [16], [26] and PP1 has been shown to localise at tight junctions in epithelial cells [13], we hypothesised that ASPP2 may dephosphorylate YAP via the recruitment of PP1. Interestingly, in MDCK cells stably expressing an shRNA against endogenous canine ASPP2, both wild type and a PP1 binding defective ASPP2 mutant (ASPP2 (RAKA)-V5) [16] displayed the same junctional pattern, suggesting that ASPP2's localisation is not dependent on its interaction with PP1 (**Figure S3A**). In agreement, both constructs could interact with endogenous Par3 (**Figure S3B**). When wild type ASPP2 and YAP were co-transfected into HEK293 cells, pYAP S127 levels decreased, whereas they remained unchanged in the presence of ASPP2 (RAKA)-V5 (**Figure 3A**). In addition, wild type ASPP2, as opposed to ASPP2 (RAKA)-V5, decreased the amount of highly phosphorylated forms of YAP (**Figure 3B**). Together, these results demonstrate that ASPP2 dephosphorylates YAP via the recruitment of PP1. Finally, a mutant of ASPP2 containing two mutations in its YAP-binding motif (Y869A/Y874A) could not interact with YAP and was consequently unable to dephosphorylate pYAP S127, indicating that ASPP2 operates via the recruitment of YAP to directly induce its dephosphorylation (**Figure 3C**). Furthermore, partially contradicting a previous report and agreeing with the lack of a WW binding motif in their proline-rich region, neither ASPP1 or iASPP could interact with YAP to induce its dephosphorylation at S127 (**Figure 3D**) [20]. Collectively, these results highlight the role of ASPP2 as a PP1 regulatory subunit by scaffolding both phosphatase and substrate to specifically dephosphorylate YAP.

### ASPP2 enhances the role of YAP as a TEAD transcription cofactor

**Figure 4:**
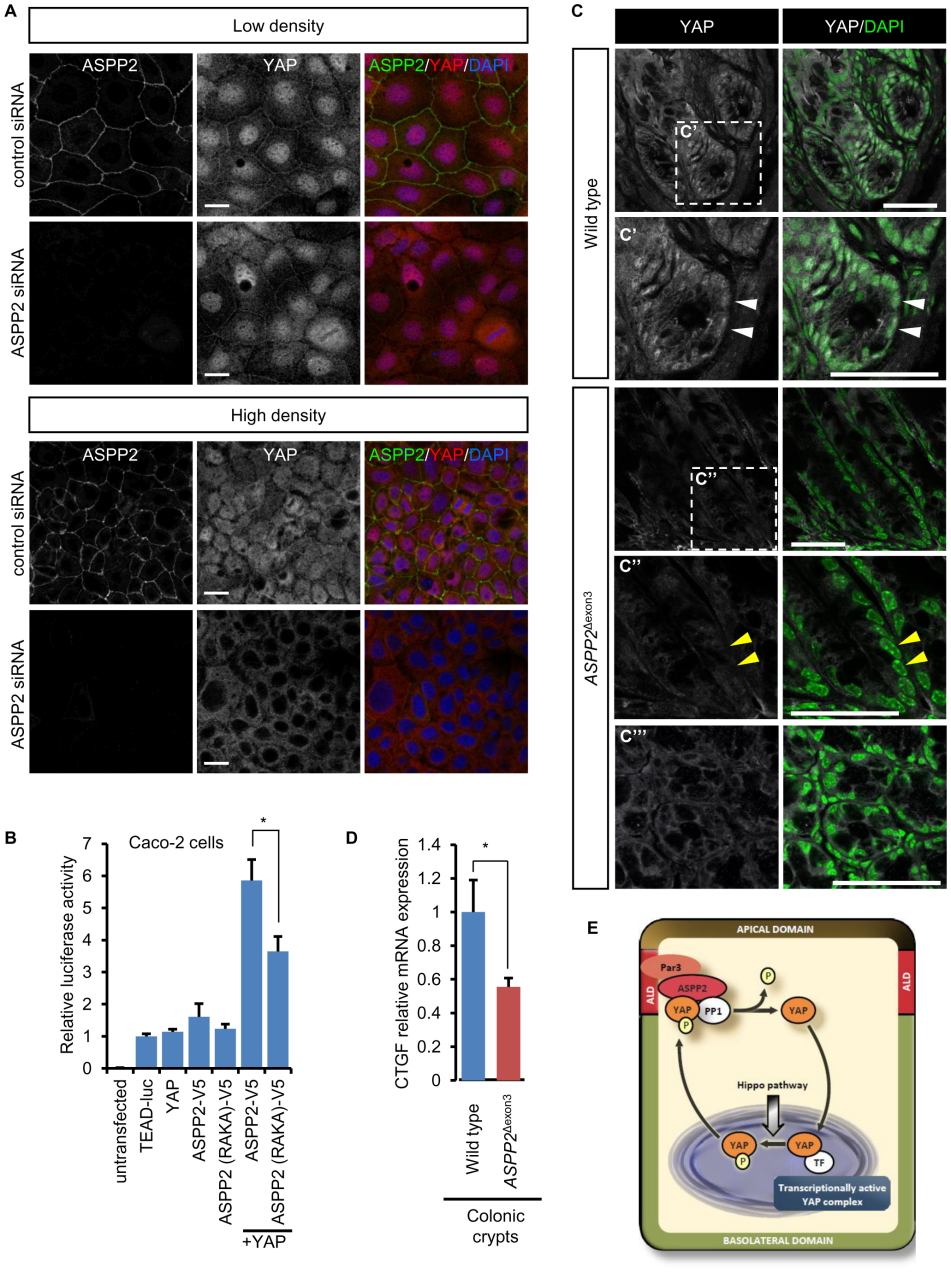
ASPP2 promotes the transcriptional activity of YAP. (**A**) Following transfection of Caco-2 cells with control or ASPP2 siRNA, the localisation of YAP and ASPP2 was analysed by immunostaining at low and high cell density. Scale bar: 20 µm. (**B**) The ability of wild type ASPP2 and ASPP2 (RAKA)-V5 to regulate TEAD-mediated transcription was analysed in a luciferase assay using the TEAD-luciferase reporter (8xGTIIC-luciferase) in Caco-2 cells. Values were obtained from three independent duplicate experiments and error bars indicate standard deviation (*: p<0.05). (**C**) YAP immunostaining of paraffin sections obtained from the colons of wild type and *ASPP2* ^Δexon3^ mice. Dashed white squares highlight magnified areas represented in the corresponding panels (C′-C′″). White arrowheads point to YAP positive nuclei in wild type crypts whereas yellow arrowheads point to nuclei devoid of YAP in *ASPP2* ^Δexon3^ crypts. Nuclei were counterstained with DAPI. Scale bars: 50 µm. (**D**) *CTGF* mRNA levels were quantified by qRT-PCR using RNA obtained from the colons of wild type (n = 3) and *ASPP2* ^Δexon3^ mice (n = 3). Error bars represent standard deviation (*: p<0.05). (**E**) Diagram representing the regulation of YAP by ASPP2 in epithelial cells. Once YAP is phosphorylated at serine 127, it can interact with ASPP2 at the apical lateral domain where ASPP2 induces its dephosphorylation via the recruitment of PP1. YAP is consequently able to relocalise to the nucleus where it can modulate TEAD transcriptional activity.

YAP phosphorylation at S127 induces its export from the nucleus to the cytoplasm via the binding to 14-3-3. In Caco-2 cells, following ASPP2 depletion, we could observe a discrete increase in cytoplasmic YAP at low cell density. However, at high cell density, ASPP2-depleted cells exhibited a striking decrease in nuclear YAP, suggesting that ASPP2, by dephosphorylating pYAP S127, may indeed promote its nuclear localisation (**Figure 4A**). To test whether this would have consequences on the function of YAP as a transcriptional co-regulator, we used a TEAD reporter in Caco-2 cells (**Figure 4B**) [3]. Interestingly, transfecting YAP or ASPP2 alone had little effect on the reporter construct, suggesting that the endogenous machinery was able to regulate the excess of exogenous YAP or ASPP2. Moreover, over-expression of ASPP2 alone, despite reducing cytoplasmic YAP expression, did not seem to increase YAP nuclear localisation (**Figure S4A**). However, co-expression of both YAP and ASPP2 lead to a robust increase in luciferase activity, suggesting that together they synergise to overcome endogenous repression. Strikingly, ASPP2 (RAKA)-V5 was less effective than wild type ASPP2, consistent with ASPP2 acting through PP1 to dephosphorylate and activate YAP. In addition to TEAD1-4, YAP regulates a wide variety of transcription factors, including p73 [27]. Furthermore, ASPP2 is known to promote the apoptotic function of p53, p63 and p73 [18], [19]. However, as opposed to the TEAD reporter, ASPP2 and YAP could not modulate the activity of a Bax-reporter in Caco-2 cells, therefore suggesting that, under unstressed conditions, they regulate TEAD rather than p53 family members (**Figure S4B**). In addition, ASPP2 did not further induce the activity of p73 on the Bax-luciferase reporter and only had a moderate effect on p53. Mutations in the YAP or PP1 binding sites of ASPP2 did not modify its ability to induce p53, suggesting that this effect is YAP independent (**Figure S4C and S4D**).

Together, these data demonstrate the role of ASPP2 in promoting the nuclear localisation and transcriptional activity of YAP *in vitro*. Recent studies have shown the importance of YAP during colonic regeneration [28], [29]. Since ASPP2 and YAP also potentially form a junctional complex in the colon (**Figure 1C**), we investigated whether ASPP2 could influence YAP activity by analysing its subcellular localisation in the colonic crypts of wild type and *ASPP2* ^Δexon3^ mice (**Figure 4C**). As previously described, YAP was predominantly nuclear in the epithelial cells of wild type colonic crypts [28]. However, in *ASPP2* ^Δexon3^ crypts, nuclei exhibited reduced YAP expression, suggesting that ASPP2 promotes the nuclear localisation of YAP *in vivo*. Interestingly, CTGF mRNA level, an established endogenous readout of YAP/TAZ activity, was markedly reduced in *ASPP2* ^Δexon3^ colonic crypts, suggesting that ASPP2 stimulates the transcriptional function of YAP *in vivo* (**Figure 4D**). Collectively our results indicate that ASPP2, at the level of the apical-lateral domain, via the recruitment of PP1, induces the dephosphorylation of junctional YAP which subsequently leads to its activation *in vitro* and *in vivo*, in colonic crypts (**Figure 4E**).

## Discussion

Our findings highlight how ASPP2, in addition to its role in regulating apicobasal polarity, acts as a scaffold for YAP and PP1 at the level of the apical-lateral domain in epithelial cells, *in vitro* and *in vivo*, therefore allowing PP1 to dephosphorylate junctional YAP.

Several regulators of apicobasal polarity such as Crumbs and AMOT have previously been associated with the regulation of YAP activity [5], [9], [30]. These proteins repress YAP by promoting the activity of the Hippo pathway. Likewise, ASPP2 can regulate apicobasal polarity by interacting with Par3 and regulating its localisation [14], [15]. However, as opposed to AMOT and Crumbs, ASPP2 represents a positive regulator of YAP and may serve to counterbalance their activity. The fact that positive and negative regulators of YAP play a role in the establishment of apicobasal polarity and are localised at tight junctions underlies the importance of the apical lateral domain in epithelial cells as a key YAP-regulating hub. The recent unveiling of the Hippo interactome strongly reinforces this view by outlining extensive links between core Hippo pathway components and regulators of apicobasal polarity including ASPP2 [6]–[8]. Our study provides mechanistic insight into how one such interaction modulates YAP activity in a biologically significant context like the colon crypt.

The localisation of ASPP2, and potentially a whole group of other Hippo regulators, at the apical-lateral domain in epithelial cells is unlikely to be coincidental. It is tempting to speculate that its position may be important to integrate upstream information via the modulation of YAP activity which would consequently translate into an adapted transcriptional program. Our data suggests that in the colon, without ASPP2, YAP signalling is repressed, suggesting there exists a strong relationship between apicobasal polarity and YAP activity. This raises the possibility that ASPP2 may be required to translate polarity cues into an adapted transcriptional program via the activation of YAP. Considering the importance of YAP during colonic regeneration, the regulation of YAP by ASPP2 may be of particular relevance during tissue regeneration in order to coordinate correct tissue architecture and growth control.

Our study explores a function of ASPP2 that may in fact play roles in the control of the architecture, growth and homeostasis of other epithelial tissues. Born *ASPP2* ^Δexon3^ mice are smaller than their wild type litter mates which would agree with ASPP2 contributing to the control of organ size [31]. Moreover, although the mechanism by which ASPP2 controls apicobasal polarity during CNS development is relatively well-understood, how it controls the proliferation of neural progenitors remains unknown [14]. YAP has been shown to control the proliferation of neural progenitors and, as a result, it is not impossible that the proliferation phenotype observed in *ASPP2* ^Δexon3^ mice may be linked to abnormal YAP activity [32].

In conclusion, ASPP2, by linking apicobasal polarity to YAP activity, represents an exciting regulator of the Hippo pathway and future studies will define whether it may become an attractive target to modulate the activity of YAP in a disease context.

## Materials and Methods

### Ethics statement

All work involving animals has been carried out under a licence issued by the Home Office and have been approved by the Home Office.

### Histology and tissue section immunostaining

The colons of 3 month old mice were dissected and washed in PBS. Tissues were kept in 10% formalin overnight. For frozen sections, the tissues were treated with 20% sucrose overnight, embedded in OCT and snap frozen in dry ice. Serial 15µm sections were obtained. Sections were then incubated in primary antibody diluted in 5% normal goat serum in PBS overnight at 4°C followed by incubation with Alexa Fluor secondary antibodies for 30 minutes. Nuclei were counterstained with DAPI (Roche). Slides were finally mounted onto coverslips.

### Immunoprecipitation and SDS-PAGE/Immunoblotting

For immunoprecipitation experiments, HEK293 cells were transfected using Lipofectamine 2000 (Life Technologies) according to the manufacturer's protocol. Caco-2, EJ, MDCK, transfected HEK293 cells and colon samples were lysed in RIPA 1% IGEPAL for SDS-PAGE/Immunoblotting or a buffer containing 50 mM Tris-HCl at pH 8, 150 mM NaCl, 1 mM EDTA, Complete Protease Inhibitor Cocktail (Roche) and 1% Triton X-100 for immunoprecipitation. Lysates were then subjected to immunoprecipitation and SDS-PAGE/Immunoblotting as previously described (Yap *et al*., 2000). Densitometry analysis was performed using ImageJ. Phos-Tag gels were prepared according to the manufacturer's protocol.

### Luciferase assay

Caco-2 cells were plated in 24-well plates and the next day transfected using Lipofectamine 2000 (Life Technologies) with the indicated constructs. The following amounts of DNA were used per well in the assay: Luciferase reporter, 200 ng; Renilla-luciferase, 10 ng; hYAP-myc, 50 to 200 ng; ASPP2-V5 and mutants, 800 ng; p73, 50 ng. PCDNA3.1 was used to keep DNA total amounts constant from one condition to another. The assay was performed 24 hours after transfection using the Dual Glo luciferase assay system (Promega) according to the manufacturer's protocol.

### Real-Time Quantitative PCR

Real-time quantitative PCR (qRT-PCR) was performed as previously described with the 7500 real-time PCR system (Applied Biosystems) using the QuantiTect SYBR Green PCR kit (Qiagen) [33]. In short, each reaction was performed in triplicate using 1 µL of cDNA in a final volume of 25 µL. The expression level of *CTGF* was analyzed based on the ΔΔCt method, with GAPDH as an internal control. The following thermal cycle was used for all samples: 15 min at 95°C; 45 cycles of 15 s at 94°C, 30 s primer-specific annealing temperatures, 1 min at 72°C. For each experiment, the threshold was set to cross a point at which realtime PCR amplification was linear.

### siRNA knockdown

siRNA oligos against human ASPP2, Lats1, Lats2, Par3, YAP and RISC-Free siRNA were purchased from Dharmacon. Cells were transfected with the indicated siRNA oligos at a final concentration of 35 nM using Dharmafect 1 reagent (Dharmacon) for 3 to 5 days, according to the manufacturer's instructions.

### Immunocytochemistry

Caco-2 cells were seeded onto coverglasses. Lipofectamine 2000 was used for transient expression of ASPP2-V5 in Caco-2 cells. MDCK cells were plated on 6.5 mm diameter Transwell filters (Corning) with a 0.4 mm pore size as previously described [14]. Once experimental procedures had been carried out, cells were fixed with 4% paraformaldehyde in PBS for 10 min and then permeabilized with 0.1% Triton X-100 in PBS for 4 min. PBS containing 2% BSA was used as a blocking solution for 20 minutes prior to incubation with primary antibodies. Primary antibodies were diluted in PBS containing 2% of BSA, and applied to cells for 40 minutes. Protein expression was detected using Alexa Fluor (1∶400, Molecular Probes) for 20 min. DAPI (Invitrogen) was used to stain nuclei (1∶2000).

### Statistical analysis

The T-test was used to calculate the statistical significance between two measurements. Differences were considered significant at a value of p≤0.05

## Supporting Information

Figure S1 (**A**) Maximum intensity projection of YAP and ASPP2 immunostaining in polarised MDCK cells. Nuclei are counterstained with DAPI. (**B**) YAP and ASPP2 immunostaining in Caco-2 cells transfected with control or YAP siRNA. Note the reduced junctional and nuclear YAP signal following YAP knockdown. (**C**) Protein sequence alignments performed using ClustalW. Left panel: amongst all ASPP family members, the YAP-binding motif is present in human ASPP2 only. Right panel: As opposed to the PP1-binding domain of ASPP2 that is conserved across evolution, the YAP-binding motif is only present in vertebrates. (**D**) Co-immunoprecipitation of ASPP2 and YAP in MDCK cells plated at different cell densities. Lysates were obtained from MDCK cells plated at various cell densities and endogenous ASPP2 was immunoprecipitated with an anti-ASPP2 mouse monoclonal antibody (DX50.13) and an anti-Gal4 mouse monoclonal antibody was used as a negative control. YAP and YAP phosphorylated at S127 were subsequently detected by SDS-Page/immunoblotting. β-tubulin was used as loading control. LD: low density; MD: medium density; HD: high density. (**E**) The localisation of YAP phosphorylated at S127 and ASPP2 was analysed by immunostaining in Caco-2 cells transfected with control or YAP siRNA. E′ and E″ are magnified views of the corresponding dashed areas. White arrowheads point to junctional YAP phosphorylated at S127.
(TIF)

Figure S2 (**A**) ASPP2 depletion leads to increased phosphorylated YAP at serine 127. ASPP2 was depleted in MDCK cells using an shRNA against canine ASPP2 and the levels of the indicated proteins were analysed by SDS-Page/immunoblotting. (**B**) SDS-Page/immunoblotting was performed on lysates obtained from the colons of wild type or *ASPP2* ^Δexon3^ mice to detect the expression levels of the indicated proteins. β-tubulin was used as a loading control.
(TIF)

Figure S3 (**A**) The localisation of ASPP2-V5 and ASPP2 (RAKA)-V5 was examined by immunostaining in MDCK cells depleted of endogenous canine ASPP2. Note the distribution of both constructs at cell-cell junctions. (**B**) The ability of iASPP-V5, ASPP2-V5 and ASPP2 (RAKA)-V5 to interact with endogenous Par3 was tested in U2OS cells. An anti-V5 mouse monoclonal antibody was used to immunoprecipitate these constructs and SDS-Page/immunoblotting was subsequently performed using the indicated antibodies. Black arrowheads point to ASPP2-V5 and iASPP-V5 respectively. White arrowheads point to different Par3 isoforms. Note that, as opposed to iASPP-V5, both ASPP2-V5 and ASPP2 (RAKA)-V5 could co-immunoprecipitate with endogenous Par3. As previously described, ASPP2 (RAKA)-V5 did not interact with endogenous PP1_α_.
(TIF)

Figure S4 (**A**) ASPP2-V5 was transfected into Caco-2 cells and its effect on endogenous YAP localisation was subsequently analysed by immunostaining. (**B**) The ability of ASPP2 and YAP to regulate transcriptional events mediated by TEAD or Bax was tested in a luciferase assay in Caco-2 cells. (**C-D**) The ability of ASPP2 to regulate the transcriptional function of p73 (B) and p53 (C) was analysed in Caco-2 cells using a Bax-luciferase reporter. Of note, ASPP2 (RAKA)-V5 and ASPP2 (Y869A/Y874A)-V5 behaved similarly to wild type ASPP2-V5 when co-expressed with p73 or p53.
(TIF)

File S1 Information on mouse models and reagents. This file contains detailed information on mouse colonies, cell lines, primary antibodies, plasmids and primers used in the study.
(DOCX)
