# Supplementary figures and images for: ASPP2 Links the Apical Lateral Polarity Complex to the Regulation of YAP Activity in Epithelial Cells
Source: PLoS One. 2014 Oct 31;9(10):e111384. doi: 10.1371/journal.pone.0111384 (PMC4216074; doi:10.1371/journal.pone.0111384)

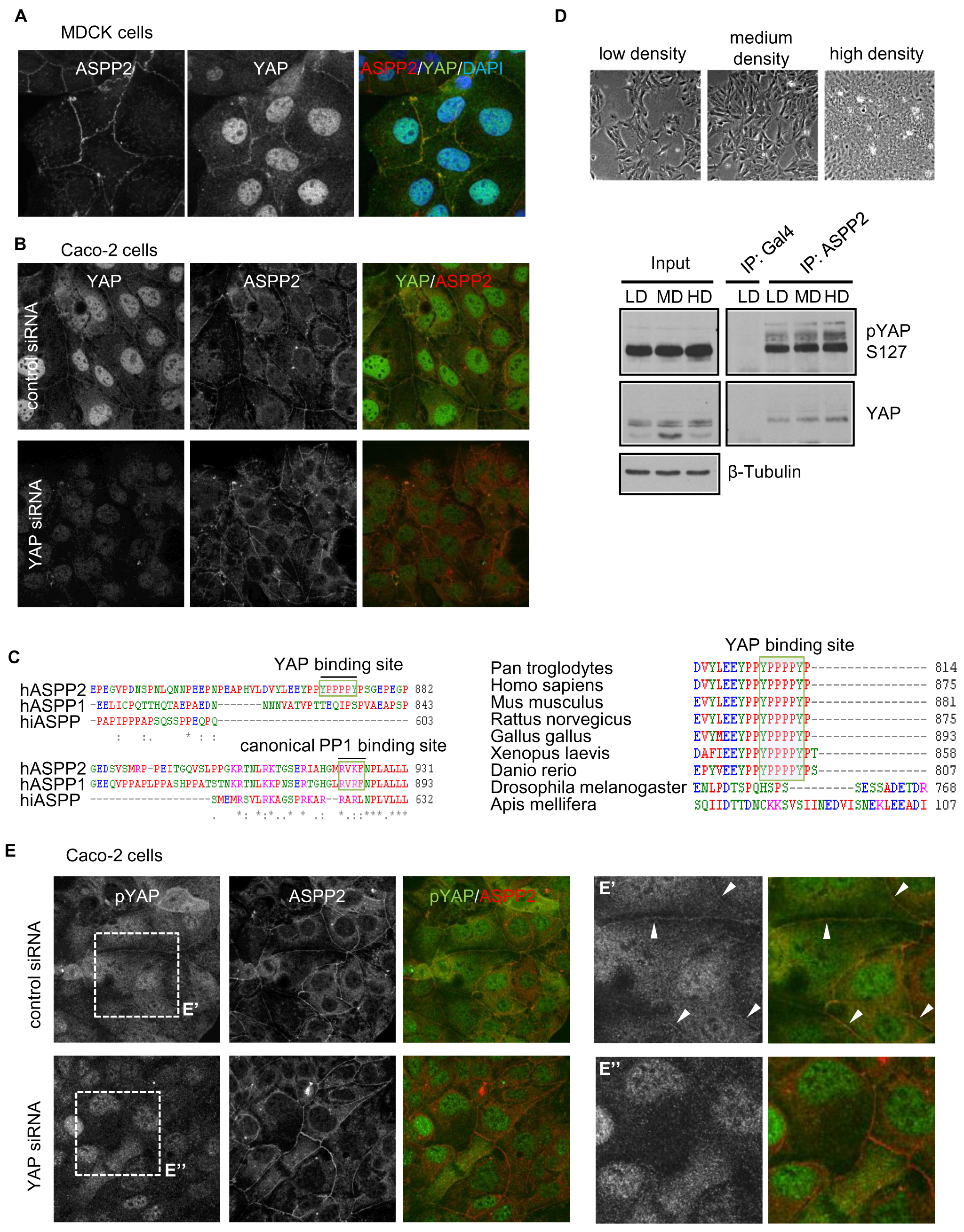

Supplement: Figure S1 — (A) Maximum intensity projection of YAP and ASPP2 immunostaining in polarised MDCK cells. Nuclei are counterstained with DAPI. (B) YAP and ASPP2 immunostaining in Caco-2 cells transfected with control or YAP siRNA. Note the reduced junctional and nuclear YAP signal following YAP knockdown. (C) Protein sequence alignments performed using ClustalW. Left panel: amongst all ASPP family members, the YAP-binding motif is present in human ASPP2 only. Right panel: As opposed to the PP1-binding domain of ASPP2 that is conserved across evolution, the YAP-binding motif is only present in vertebrates. (D) Co-immunoprecipitation of ASPP2 and YAP in MDCK cells plated at different cell densities. Lysates were obtained from MDCK cells plated at various cell densities and endogenous ASPP2 was immunoprecipitated with an anti-ASPP2 mouse monoclonal antibody (DX50.13) and an anti-Gal4 mouse monoclonal antibody was used as a negative control. YAP and YAP phosphorylated at S127 were subsequently detected by SDS-Page/immunoblotting. β-tubulin was used as loading control. LD: low density; MD: medium density; HD: high density. (E) The localisation of YAP phosphorylated at S127 and ASPP2 was analysed by immunostaining in Caco-2 cells transfected with control or YAP siRNA. E′ and E″ are magnified views of the corresponding dashed areas. White arrowheads point to junctional YAP phosphorylated at S127. (TIF) [file pone.0111384.s001.tif]

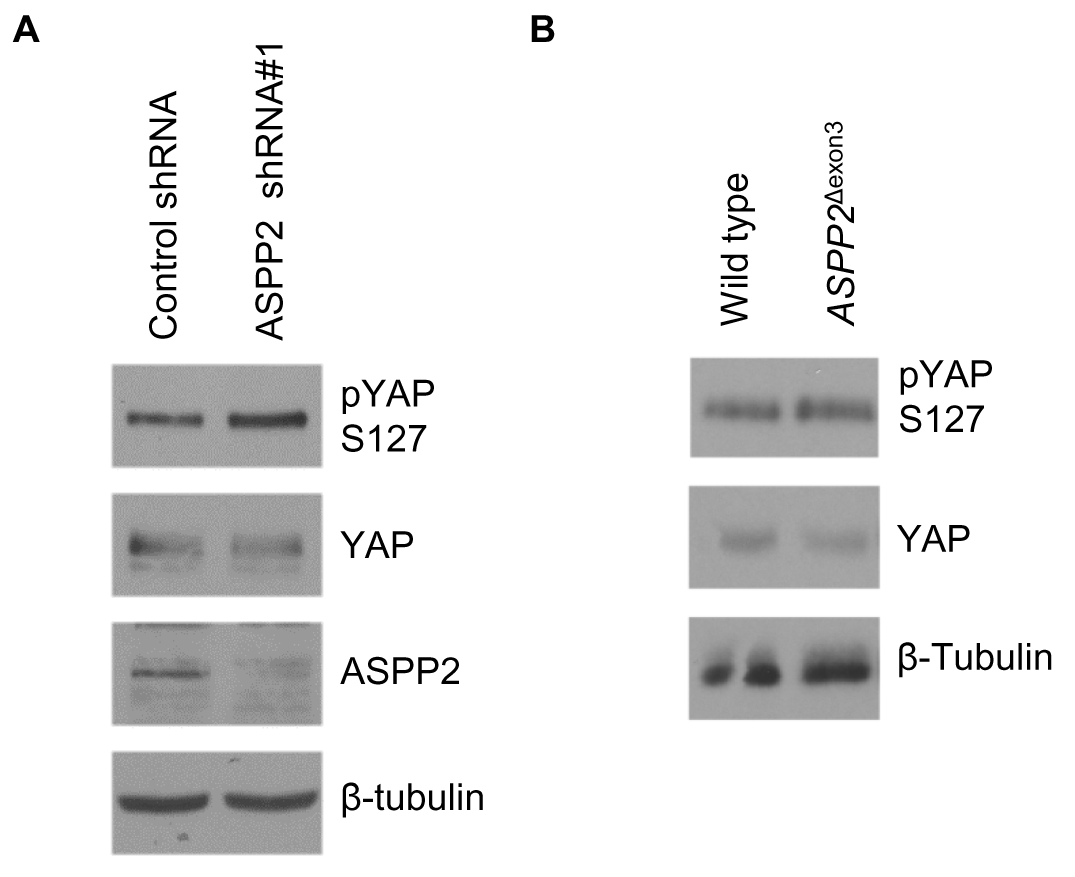

Supplement: Figure S2 — (A) ASPP2 depletion leads to increased phosphorylated YAP at serine 127. ASPP2 was depleted in MDCK cells using an shRNA against canine ASPP2 and the levels of the indicated proteins were analysed by SDS-Page/immunoblotting. (B) SDS-Page/immunoblotting was performed on lysates obtained from the colons of wild type or ASPP2 Δexon3 mice to detect the expression levels of the indicated proteins. β-tubulin was used as a loading control. (TIF) [file pone.0111384.s002.tif]

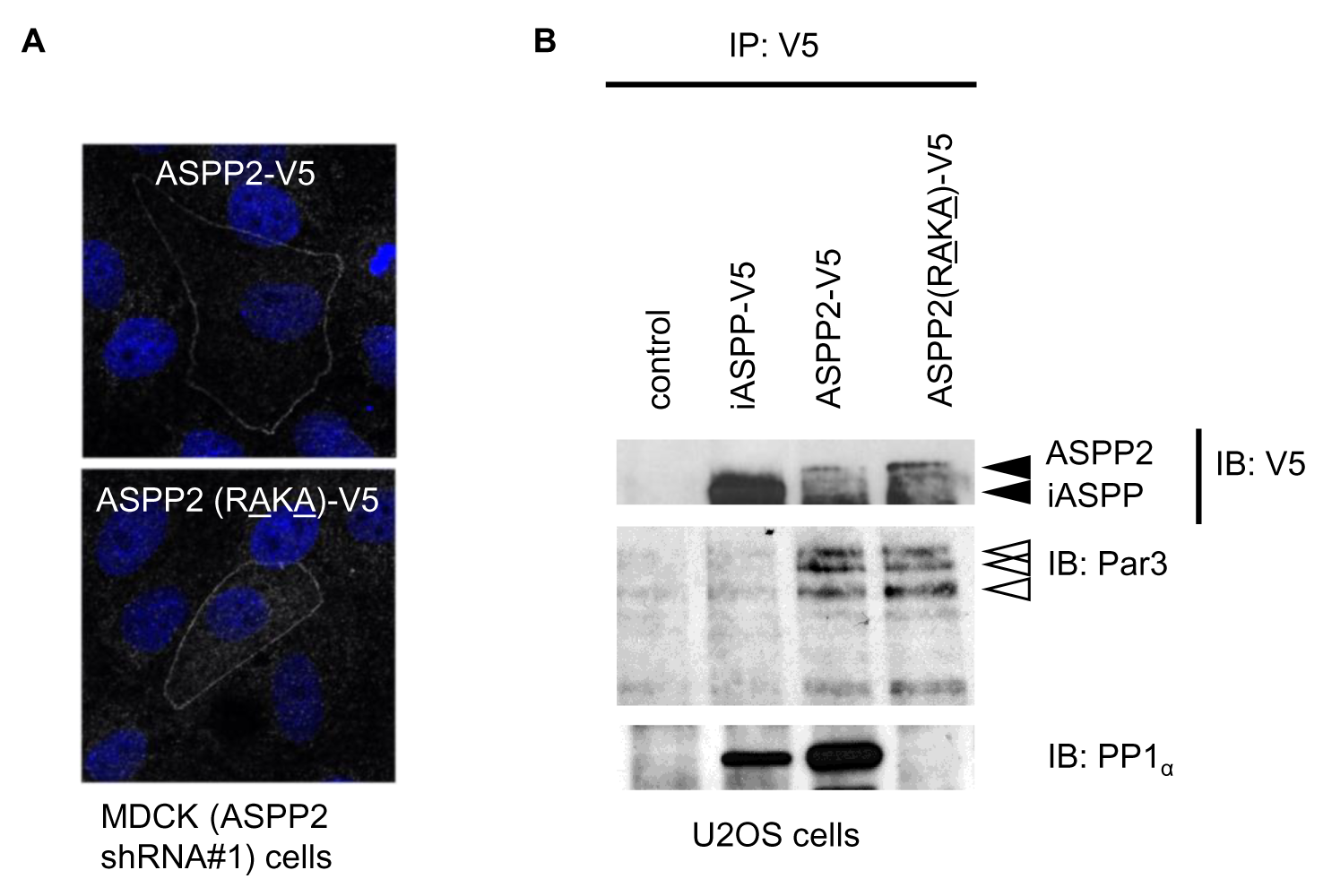

Supplement: Figure S3 — (A) The localisation of ASPP2-V5 and ASPP2 (RAKA)-V5 was examined by immunostaining in MDCK cells depleted of endogenous canine ASPP2. Note the distribution of both constructs at cell-cell junctions. (B) The ability of iASPP-V5, ASPP2-V5 and ASPP2 (RAKA)-V5 to interact with endogenous Par3 was tested in U2OS cells. An anti-V5 mouse monoclonal antibody was used to immunoprecipitate these constructs and SDS-Page/immunoblotting was subsequently performed using the indicated antibodies. Black arrowheads point to ASPP2-V5 and iASPP-V5 respectively. White arrowheads point to different Par3 isoforms. Note that, as opposed to iASPP-V5, both ASPP2-V5 and ASPP2 (RAKA)-V5 could co-immunoprecipitate with endogenous Par3. As previously described, ASPP2 (RAKA)-V5 did not interact with endogenous PP1α. (TIF) [file pone.0111384.s003.tif]

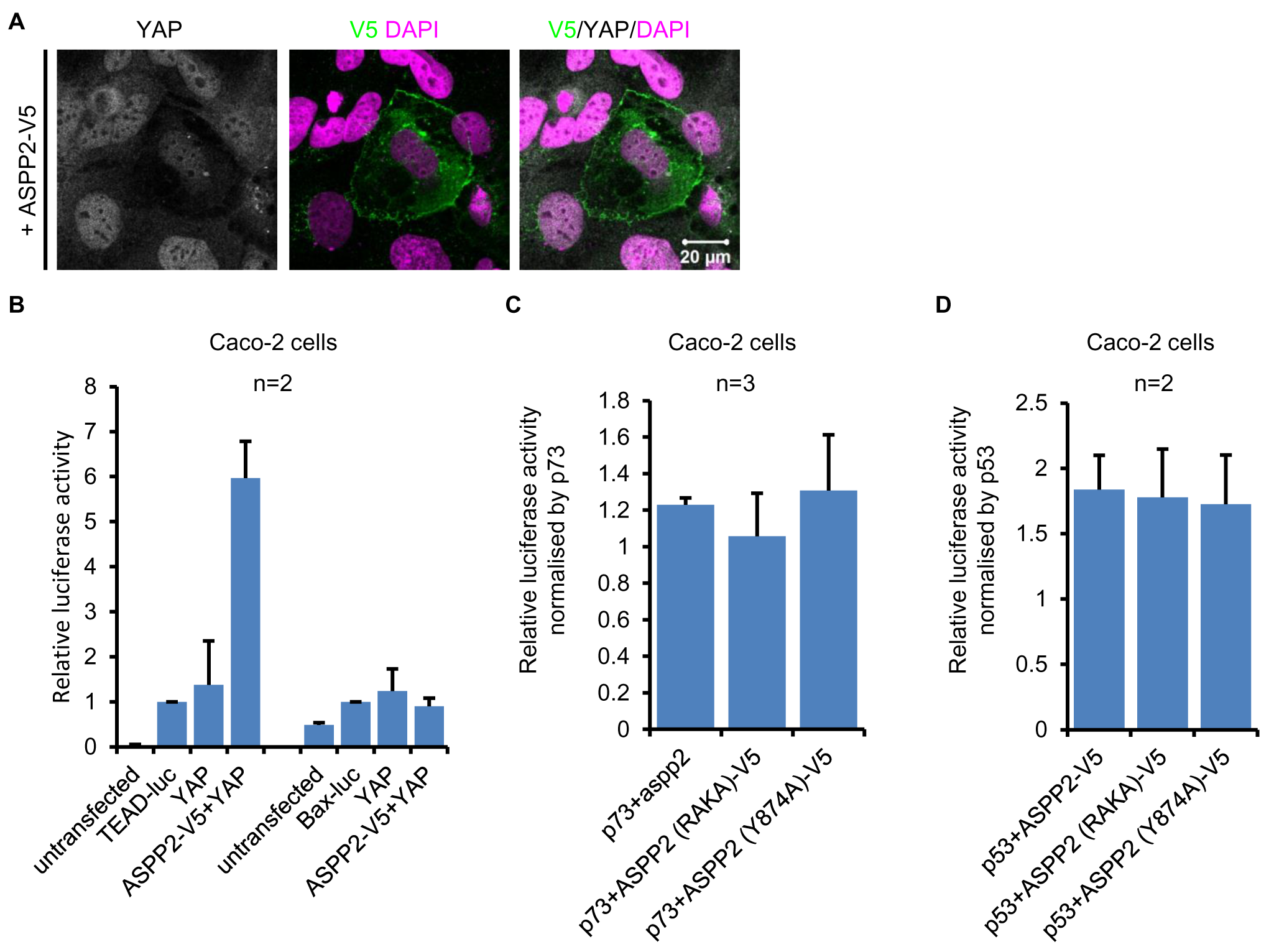

Supplement: Figure S4 — (A) ASPP2-V5 was transfected into Caco-2 cells and its effect on endogenous YAP localisation was subsequently analysed by immunostaining. (B) The ability of ASPP2 and YAP to regulate transcriptional events mediated by TEAD or Bax was tested in a luciferase assay in Caco-2 cells. (C-D) The ability of ASPP2 to regulate the transcriptional function of p73 (B) and p53 (C) was analysed in Caco-2 cells using a Bax-luciferase reporter. Of note, ASPP2 (RAKA)-V5 and ASPP2 (Y869A/Y874A)-V5 behaved similarly to wild type ASPP2-V5 when co-expressed with p73 or p53. (TIF) [file pone.0111384.s004.tif]
