# Supplementary material for: ASPP2 Links the Apical Lateral Polarity Complex to the Regulation of YAP Activity in Epithelial Cells
Source: PLoS One. 2014 Oct 31;9(10):e111384. doi: 10.1371/journal.pone.0111384 (PMC4216074; doi:10.1371/journal.pone.0111384)
Supplement: File S1 — Information on mouse models and reagents. This file contains detailed information on mouse colonies, cell lines, primary antibodies, plasmids and primers used in the study. (DOCX) [file pone.0111384.s005.docx]

Mouse colonies

*ASPP2*^Δexon3^ mice were generated in a mixed C57BL/6Jx129SvJ background and backcrossed in a Balb/c background for 9 generations (Vives et al., 2006). *ASPP2*^Δexon3^ mice were genotyped as previously described using the following primers: 5’-CTCCACCCCAGGAAATTACA-3’ (intron3), 5’-CGGTTTGGAAGTCAAAGGAA-3’ (exon 3) and 5’-GGACCGCTATCAGGACATA-3’ (neomycin resistance gene). *ASPP2*^Δ3loxP-CreER^ mice in which exon3 of *ASPP2* can be deleted following tamoxifen intraperitoneal injections were generated as previously described (Sottocornola et al., 2010). *ASPP2*^Δ3loxP-CreER^ mice were genotyped using the following primers: ASPP2, 5’-CGGTTTGGAAGTCAAAGGAA-3’ and 5’-TCAATGTGTCCAGCACCCTA-3’; LoxP sites, 5’-TGGACAGGCTGAGCTGTTAG-3’ and 5’-ACACTTTCACCAACCCTAGGTC-3’; Cre, 5’-CATTTGGGCCAGCTAAACAT-3’ and 5’-ATTCTCCCACCGTCAGTACG-3’.

**Cell Lines**

All cells were grown in Dulbecco’s modified Eagle’s medium containing 10% fetal calf serum, penicillin, and streptomycin at 37°C in a 5% CO2 atmosphere incubator. HEK293T cells were obtained from Thermo scientific (HCL4517) and MDCK.2 cells were obtained from ATCC (CRL-2936). Stable cell lines derived from Caco-2, MDCK and EJ cells expressing the indicated constructs were established using the following protocol (see plasmids for more details about the constructs). Briefly, HEK293T cells were transfected with plasmids encoding VSV-G and Gag-Pol, as well as the wild type or mutant YAP-expressing plasmid or ASPP2 shRNA construct using Lipofectamine reagents. 24 hr and 48 hr post-transfection, media containing viral particles were collected, filtered through a 0.45 µm membrane, and Caco-2, MDCK or EJ cells were incubated with 500 µL of viral preparation supplemented with 6 µg/mL Polybrene (Sigma). The medium was replaced after 6hr. After 24hr cells were placed under puromycin selection (2mg/mL).

**Primary antibodies**

| Antigen | Name | Source | Application |
| --- | --- | --- | --- |
| mASPP2 | DX50.13 | Mouse monoclonal (X Lu’s lab) | IP |
| mASPP2 | S32 | Rabbit polyclonal (X Lu’s lab) | WB |
| hASPP2 | DX54.10 | Mouse monoclonal (X Lu’s lab) | ICC |
| hASPP2 | HPA021603 | Rabbit polyclonal, Sigma | WB/IHC |
| hASPP2 | 19 | Mouse monoclonal, Santa Cruz Biotechnologies | WB/ICC |
| ASPP1 | LX54.2 | Mouse monoclonal (X Lu’s lab) | WB |
| iASPP | LX49.3 | Mouse monoclonal (X Lu’s lab) | WB |
| YAP | H125 | Rabbit polyclonal, Santa Cruz Biotechnologies | WB/ICC/IHC |
| YAP | YAP Antibody | Rabbit monoclonal, Cell signalling | WB |
| pYAPS127 | Phospho-YAP (Ser127) | Rabbit polyclonal, Cell Signaling | WB/ICC |
| TAZ | H70 | Rabbit polyclonal, Santa Cruz Biotechnologies | WB |
| Par3 | 07-330 | Rabbit polyclonal, Millipore | ICC/WB |
| Lats1 | C66B5 | Rabbit monoclonal, Cell Signaling | WB |
| Lats2 | NB200-199 | Rabbit polyclonal, Novus Biologicals | WB |
| PP1_α_ | C-19 | Goat polyclonal, Santa Cruz Biotechnologies | WB |
| β-Tubulin | TUB 2.1 | Mouse monoclonal, Abcam | WB |
| Myc | 9E10 | Mouse monoclonal (X Lu’s lab) | IP/ICC/WB |
| Myc | Myc-Tag Antibody | Rabbit polyclonal, Cell signaling | WB |
| V5 | Ab15828 | Rabbit polyclonal, Abcam | WB |
| V5 | SV5-Pk1 | Mouse monoclonal, Serotec | IP/ICC |
| Flag | F1804 | Mouse monoclonal, Sigma | IP/ICC |
| Gal4 | C10 | Mouse monoclonal, Santa Cruz Biotechnologies | IP |

WB: western blot; IP: immunoprecipitation; ICC: immunocytochemistry; IHC: immunohistochemistry

**Plasmids**

| Name | Vector | Information | Tag | Source |
| --- | --- | --- | --- | --- |
| ASPP2-V5 | pcDNA3.1 |  | His, V5 | X Lu’s laboratory |
| iASPP-V5 | pcDNA3.1 |  | His, V5 | X Lu’s laboratory |
| ASPP2 (RAKA)-V5 | pcDNA3.1 |  | His, V5 | X Lu’s laboratory |
| ASPP2 (Y869A/Y874A)-V5 | pcDNA3.1 |  | His, V5 | X Lu’s laboratory |
| 8xGTIIC-luciferase |  | S Piccolo’s laboratory | - | Addgene |
| Bax-luciferase |  |  | - | Bergamaschi et al., 2004 |
| hYAP-myc | pQCXIH | KL Guan’s laboratory | myc | Addgene |
| hYAP-5SA-myc | pQCXIH | KL Guan’s laboratory | myc | Addgene |
| hYAP-S127A-Flag | pQCXIH | KL Guan’s laboratory | flag | Addgene |
| P73 |  |  |  | Bergamaschi et al., 2004 |

To design the ASPP2 (Y869A/Y874A)-V5 mutant, mutations were introduced into a vector carrying V5-tagged wild type ASPP2 using site-directed mutagenesis. First the Y874A mutation was introduced using 5’ -CCACCAGCCCCATCTGGGGAGC-3’and 5’-GATGGGGCTGGTGGGGGTGGGTA-3’. Then the Y869A mutation was introduced using 5’-GAGTACCCTCCAGCCCCACCCC-3’ and 5’-TATGGTGGGGGTGGGGCTGGAGG-3’. First, the template was amplified by PCR using the mutagenesis primers and Phusion Hot Start II polymerase (Thermo Scientific), the reaction mixture then digested by DpnI treatment for 3h and purified (Geneflow Q-spin PCR purification kit). The purified DNA was used to transform Gold competent cells (Bioline) by heat-shock transformation. Colonies carrying the correctly mutated construct were identified by isolating the plasmid (Qiagen Miniprep kit) and Sanger-sequencing the relevant region of ASPP2.

Sequence of the primers used to generate the shRNA construct for ASPP2 and control were described in Cong et al. 2010. Canine ASPP2 and control shRNA primers were cloned in pSuper-Retro-puro vector.

**Primers for qRT-PCR and RT-PCR**

| Name | Sequence (5’-3’) |
| --- | --- |
| *mGADPH* F | TGTCAGCAATGCATCCTGCA |
| *mGADPH* R | TGTATGCAGGGATGATGTTC |
| *mCTGF* F | CCACCCGAGTTACCAATGAC |
| *mCTGF* R | GTGCAGCCAGAAAGCTCA |
| *LP2 (mASPP2exon 3 forward)* | AAAATGATGCCGCATGTTCCT |
| *RP7 (mASPP2exon 3 reverse)* | TTCTCCTGTTCAGCAGCTTG |
